# Twice-daily insulin degludec/insulin aspart effectively improved morning and evening glucose levels and quality of life in patients previously treated with premixed insulin: an observational study

**DOI:** 10.1186/s13098-018-0366-x

**Published:** 2018-08-16

**Authors:** Kanta Fujimoto, Toshio Iwakura, Megumi Aburaya, Naoki Matsuoka

**Affiliations:** 1 Department of Diabetes and Endocrinology, Kobe City Medical Center General Hospital, 2-1-1 Minatojima-minamimachi, Chuo-ku, Kobe, 6500047 Japan; 2 Department of Pharmacy, Kobe City Medical Center General Hospital, Kobe, Japan

**Keywords:** Insulin degludec/insulin aspart, Glucose variability, Quality of life, Type 2 diabetes

## Abstract

**Background:**

Previous studies comparing insulin degludec/insulin aspart (IDegAsp) with premixed insulin twice daily among insulin users with type 2 diabetes have not thoroughly investigated differences in the glucose variability and psychological evaluations related to insulin regimen changes. We investigated changes in the daily and day-to-day glucose variability and quality of life (QOL) related to insulin use in patients with type 2 diabetes during a switch from premixed insulin preparations comprising either human insulin (BHI30) or insulin aspart (BIAsp30) to IDegAsp twice daily.

**Methods:**

In this prospective observational study, 22 subjects (BHI30:BIAsp30 = 12:10) self-measured their blood glucose levels every morning, and before and after all meals each week. Premixed insulin was administered for the first 2 months, followed by IDegAsp for the next 2 months. Efficacy measures were evaluated during the last month or last day of both phases.

**Results:**

The mean blood glucose levels (175.5 vs. 163.0 mg/dL; *P *= 0.004) and the M-values (53.9 vs. 27.6; *P *= 0.049) were significantly lower in the IDegAsp phase. However, no differences in the standard deviations of morning fasting glucose levels were observed between phases (premixed vs. IDegAsp, 20.0 vs. 19.3 mg/dL; *P *= 0.343). Compared to the premixed phase, the before-breakfast (145.3 vs. 126.0 mg/dL; *P *< 0.001), after-breakfast (190.3 vs. 170.7 mg/dL; *P *= 0.001), before-dinner (153.0 vs. 140.1 mg/dL; *P * = 0.007), and after-dinner glucose levels (198.7 vs. 181.4 mg/dL; *P *= 0.018) were lower in the IDegAsp phase. However, the before-lunch (150.8 vs. 148.2 mg/dL; *P  *= 0.329) and after-lunch glucose levels (214.7 vs. 211.4 mg/dL; *P *= 0.308) did not significantly differ between phases. Regarding QOL, the total and therapy-related feeling Insulin Therapy Related-QOL (ITR-QOL) questionnaire scores favored IDegAsp, as did the ITR-QOL at Night questionnaire subscale score of glycemic control before breakfast.

**Conclusions:**

Although the day-to-day variability of morning fasting glucose levels did not change, switching to IDegAsp improved daily glucose level variability, the morning and evening glucose control and QOL among patients treated with premixed insulin.

*Trial registration* University Hospital Medical Information Network Clinical Trials Registry, UMIN000021939. Prospectively registered 18 April 2016

## Background

Basal insulin is a recommended insulin regimen for patients with type 2 diabetes according to the guidelines of Western countries [1]. However, nocturnal hypoglycemia with basal insulin has emerged due to its features, and additional bolus insulin may be required to achieve near-normal glycemic levels. As the basal-bolus insulin regimen is complex and requires multiple daily injections, the combination of basal/intermediate and prandial insulin can potentially reduce the burden on patients. The currently available premixed insulin therapies, which comprise a 3:7 fast: intermediate ratio consisting of either human insulin (BHI30) or insulin aspart (BIAsp30), are widely used especially in Asia [2]. However, the protaminated fraction of premixed insulin interacts with the soluble fraction, resulting in a “shoulder effect” that prolongs the effect of the soluble fraction [3], and they are often incorrectly resuspended by patients [4].

Insulin degludec/insulin aspart (IDegAsp) is a soluble combination of components that provide separate basal and prandial effects without requiring resuspension [5]. In previous studies comparing IDegAsp with BIAsp30 twice daily among insulin users with type 2 diabetes, IDegAsp was superior in terms of maintaining a low fasting plasma glucose level with a low daily insulin dose [6, 7]. However, patient adherence and follow-up intervals differed between these clinical trials and real-world settings, suggesting that it is unclear whether these results are applicable to general care. Additionally, these studies did not thoroughly investigate differences in the daily and day-to-day glucose variability between therapies. Furthermore, few psychological evaluations specifically related to insulin therapy in the IDegAsp regimen have been reported.

In this study, we investigated changes in the daily and day-to-day glucose variability and quality of life (QOL) related to insulin use in patients with type 2 diabetes in a real-world setting who switched from BHI30 or BIAsp30 twice daily to IDegAsp twice daily.

## Methods

### Patients

We enrolled adult patients with type 2 diabetes who attended Kobe City Medical Center General Hospital, Kobe, Japan and had used premixed insulin therapy (BHI30 or BIAsp30) twice daily for more than 6 months before the trial. We excluded patients who had changed anti-diabetic therapies during the 3 months prior to the trial initiation; with known allergies to IDegAsp, insulin degludec, or insulin aspart; with diabetic retinopathy at a higher than moderate non-proliferative stage; those receiving corticosteroids; or those with advanced cancers.

### Study design

Between April 2016 and March 2017, this prospective 4-month pilot study was conducted to assess the impact of switching from premixed insulin therapy to IDegAsp on glycemic variability and QOL. Upon study enrollment, the subjects were asked about the degree of habitual insulin resuspension before injection using the following 3-grade scale: low (0–3 times), intermediate (4–8 times), and high (≥ 9 times). The patients were then asked to record their self-measured blood glucose (SMBG) levels every morning using the same models of Medisafe FIT (Terumo Co. Ltd., Tokyo, Japan), as well as before and 2 h after all meals each week during the 4-month study period. The SMBG levels were used to calculate the study endpoint parameters. During the study, the premixed insulin therapies were continued as before and were administered before breakfast and dinner. The patients self-titrated their doses using the algorithm described below to achieve a fasting blood glucose level of 70–130 mg/dL based on the SMBG levels in the first 2 months. To avoid nocturnal hypoglycemia and optimize glycemic control, physicians adjusted the insulin doses and balance 1 month after the study began.

**Fig. 1:**
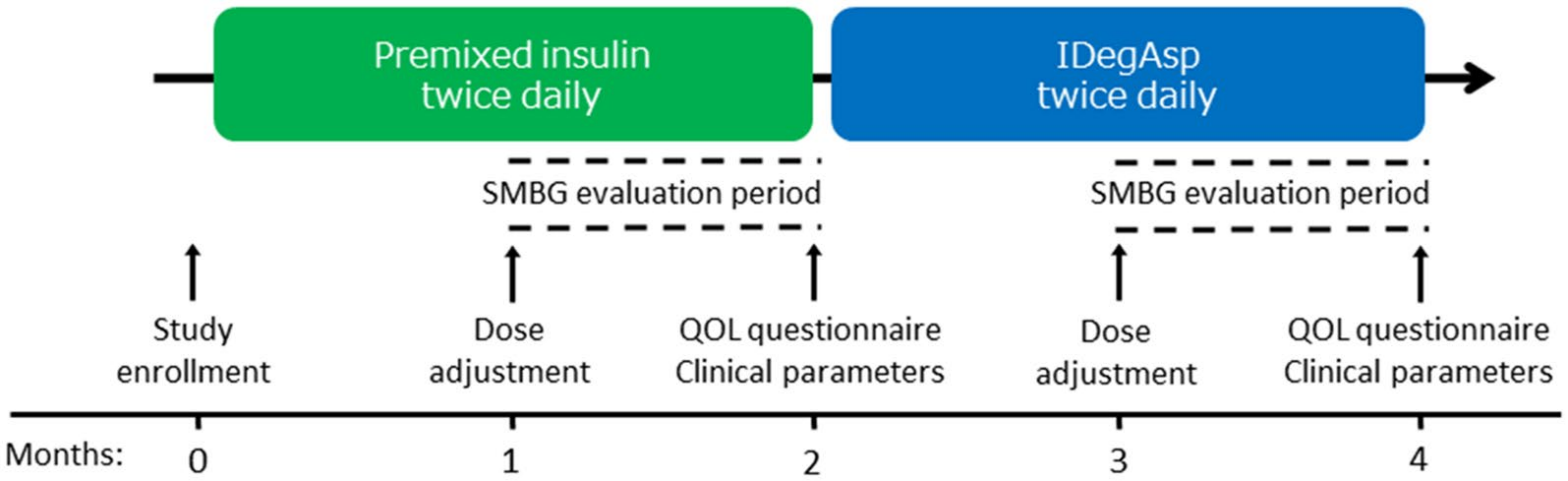
Study design: a prospective observational study. *IDegAsp* insulin degludec/insulin aspart, *QOL* quality of life

After a 2-month premixed insulin phase, the insulin therapy was switched to IDegAsp twice daily before breakfast and dinner. The insulin doses were reduced by 10–20% relative to the premixed phase with the same distribution as the previous insulin balance, after which the insulin doses were titrated similarly for the next 2 months, with an adjustment of dose and balance after 1 month by a physician. The doses of other anti-diabetic medicines and statins, diet therapies, and exercise were continued throughout the study period. The study strategy is illustrated in Fig. 1. The insulin titration algorithm used in both phases is described below. If the before-breakfast SMBG level exceeded 130 mg/dL or 180 mg/dL for 2 consecutive days, the morning and dinner insulin doses were increased by 10% and 20%, respectively. When the SMBG level at any time was < 70 mg/dL, the insulin doses in the morning and at dinner were both reduced by 10%.

The study protocol was approved by the ethics committee of Kobe City Medical Center General Hospital, where the study was conducted. The study was performed in accordance with the tenets of the Declaration of Helsinki. All study participants provided written informed consent prior to enrollment. The study was registered with the University Hospital Medical Information Network Clinical Trials Registry (UMIN000021939).

### Study endpoints

The efficacy measures included the mean glucose levels at all SMBG time points, the standard deviation (SD) of the fasting blood glucose level in the morning, M-value [8], Insulin Therapy Related Quality of Life (ITR-QOL) scores [9], and Insulin Therapy-Related Quality of Life at Night (ITR-QOLN) scores [10]. These measures were obtained during the last month or last day of both phases to avoid carryover effects. The primary endpoints were the SD of the fasting blood glucose level in the morning and the M-value. We performed sub-analyses stratified by the type of insulin therapy and renal function to address the possible effects of these factors on the results. The secondary endpoints included the mean glucose level, hypoglycemia frequency, ITR-QOL score, and ITR-QOLN score. SMBG levels < 70 mg/dL in both measurements obtained in the last month were reported to assess the frequency of hypoglycemia.

The ITR-QOL is a QOL questionnaire intended to determine the effects of insulin therapy on daily life. This instrument comprises 23 questions scored on a five-point scale (1–5) and contains four subscales: social activities, physical functioning, daily activities, and feelings about insulin treatment. The ITR-QOLN was further established to measure the QOL in relation to overnight insulin therapy and includes 21 questions scored on a seven-point scale (0–6) divided into four subscales: anxiety before sleep, disturbances during sleep, glycemic control before-breakfast, and well-being. Because lower scores on item number 23 of the ITR-QOL and items 1–18 of the ITR-QOLN indicate a better QOL, responses to these questions were converted to an inverse scale (1–5 and 0–6, respectively). Accordingly, the maximum ITR-QOL and ITR-QOLN scores were 115 and 126 points, respectively, and higher total scores on both QOL questionnaires implied a better QOL.

### Statistical analysis

Sample size calculations were based on feasibility with clinical considerations and the results from a previous comparison of IDegAsp and IAsp30 [6]. This study assumed a detectable difference between therapies of 9 mg/dL and an SD of 10 mg/dL for “the fasting blood glucose SD level in the morning (primary endpoint).” Using a two-sided significance level of 0.05 and power of 80% with an estimated dropout rate of 15%, we estimated a required sample size of 23 participants. Except for baseline characteristics (presented as mean ± SDs), data are presented as means ± standard errors or medians and interquartile ranges according to the distribution of the data. The effect sizes between therapies are presented as the standardized effect size of the least squared mean with bias-correction (using Hedges’ g). For statistical analyses, the study endpoints were evaluated using the paired *t*-test or Wilcoxon signed-rank test for non-normally distributed data. The influences of the degree of resuspension on parameter changes were assessed using analyses of variance followed by Tukey’s honest significant difference test for multiple comparisons. Pearson’s correlation analysis was used to evaluate the correlation between the variables. The analyses were conducted using JMP 13 software (SAS Institute Inc., Cary, NC, USA). A *P*-value of < 0.05 was considered statistically significant.

## Results

Twenty-two (prior insulin BHI30:BIAsp30 = 12:10) of the 24 patients enrolled in this study completed the study; two patients were unwilling to continue. The baseline characteristics of these 22 subjects are shown in Table 1. The degree of insulin resuspension before use varied from low (n = 6, 28%) and intermediate (n = 8, 36%) to high (n = 8, 36%).

**Table 1:** Baseline characteristics of the subjects with type 2 diabetes at study enrollment

Parameter	Patients (n = 22)
Age (years)	68.0 ± 9.9
Male, n (%)	15 (68)
BMI (kg/m^2^)	23.9 [22.8, 25.4]
Duration of diabetes (years)	18.6 ± 9.2
Duration of insulin therapy (years)	9.2 ± 5.9
HbA1c (%)	7.7 ± 0.6
Fasting plasma glucose (mg/dL)	145.2 ± 24.3
Fasting serum C-peptide (ng/mL)	1.18 [0.85, 1.58]
eGFR (mL/min/1.73 m^2^)	61.8 ± 15.0
Diabetic complications, n (%)	
Retinopathy	5 (23)
Nephropathy	7 (32)
Neuropathy	8 (36)
Prior premixed insulin, n (%)	
BHI30	12 (55)
BIAsp30	10 (45)
Oral antidiabetic agents, n (%)	
Sulfonylurea	1 (5)
Metformin	4 (18)
αGI	3 (14)
DPP-4 inhibitor	10 (46)
SGLT-2 inhibitor	1 (5)
Statins, n (%)	13 (59)

Data are presented as means ± SD, median and interquartile ranges, or n (%)

*BMI* body mass index, *HbA1c* glycated hemoglobin, *eGFR* estimated glomerular filtration rate, *BHI30* human insulin 30%/neutral protamine Hagedorn insulin 70%, *BIAsp30* insulin aspart 30%/protaminated insulin aspart 70%, *αGI* α-glucosidase inhibitor, *DPP-4* dipeptidyl peptidase-4, *SGLT-2* sodium glucose cotransporter-2

Changes in efficacy and the clinical parameters are shown in Table 2. Although the mean blood glucose level and M-value were significantly lower during the IDegAsp phase, there were no significant between-phase differences in the SDs of the morning fasting blood glucose and glycated hemoglobin levels. A habitually “high” degree of resuspension had a better influence in terms of reducing the SD of morning fasting blood glucose, compared to other degrees of resuspension (analysis of variance: high, − 5.3 mg/dL; intermediate, + 3.5 mg/dL; high, 0.0 mg/dL, *F*_(2, 19)_ = 3.70, *P *= 0.044). Although we observed a reduction in the mean evening insulin dose, the mean morning insulin dose, body weight, and hypoglycemia frequency were identical in both phases.

**Table 2:** Changes in the efficacy measures and clinical parameters during the study

Parameter	Premixed insulin (n = 22)	IDegAsp (n = 22)	Effect size of LS mean difference (95% CI)	*P*-value
SD of morning fasting glucose (mg/dL)	20.0 ± 1.9	19.3 ± 1.3	− 0.09 (− 0.68, 0.50)	0.686
M-value	53.9 [24.5, 73.3]	27.6 [13.1, 69.2]	–	0.049
HbA1c (%)	7.68 ± 0.13	7.50 ± 0.16	− 0.26 (− 0.85, 0.33)	0.066
Mean blood glucose (mg/dL)	175.5 ± 5.7	163.0 ± 5.6	− 0.46 (− 1.06, 0.14)	0.004
Morning insulin dose (U)	7.91 ± 0.70	8.05 ± 0.78	0.04 (− 0.55, 0.63)	0.576
Evening insulin dose (U)	8.23 ± 0.76	7.77 ± 0.72	− 0.13 (− 0.72, 0.46)	0.047
Hypoglycemia (episodes per month)	0.0 [0.0, 0.0]	0.0 [0.0, 0.0]	–	0.688
Body weight (kg)	64.2 ± 2.2	63.8 ± 2.1	− 0.04 (− 0.63, 0.55)	0.294
eGFR (mL/min/1.73 m^2^)	61.8 ± 3.2	62.6 ± 3.8	0.05 (− 0.54, 0.64)	0.481
Triglyceride (mg/dL)	115.8 ± 14.9	118.2 ± 16.9	0.03 (− 0.56, 0.62)	0.760
HDL cholesterol (mg/dL)	53.8 ± 2.1	52.8 ± 2.5	− 0.09 (− 0.68, 0.50)	0.063
LDL cholesterol (mg/dL)	92.2 ± 4.9	92.1 ± 5.0	0.00 (− 0.60, 0.59)	0.828
Aspartate transaminase (IU/L)	18.5 [17.0, 22.3]	18.0 [17.0, 21.3]	–	0.155
Alanine aminotransferase (IU/L)	16.5 [13.0, 24.0]	17.0 [13.8, 26.0]	–	0.801
γ-glutamyl transpeptidase (IU/L)	21.5 [17.5, 52.8]	23.0 [16.0, 58.0]	–	0.814

Data are presented as mean ± standard error or effect sizes of LS mean differences with bias corrected

*IDegAsp* insulin degludec/insulin aspart, *LS* least square, *CI* confidence interval, *HbA1c* glycated hemoglobin, *eGFR* estimated glomerular filtration rate, *HDL* high density lipoprotein, *LDL* low density lipoprotein

**Fig. 2:**
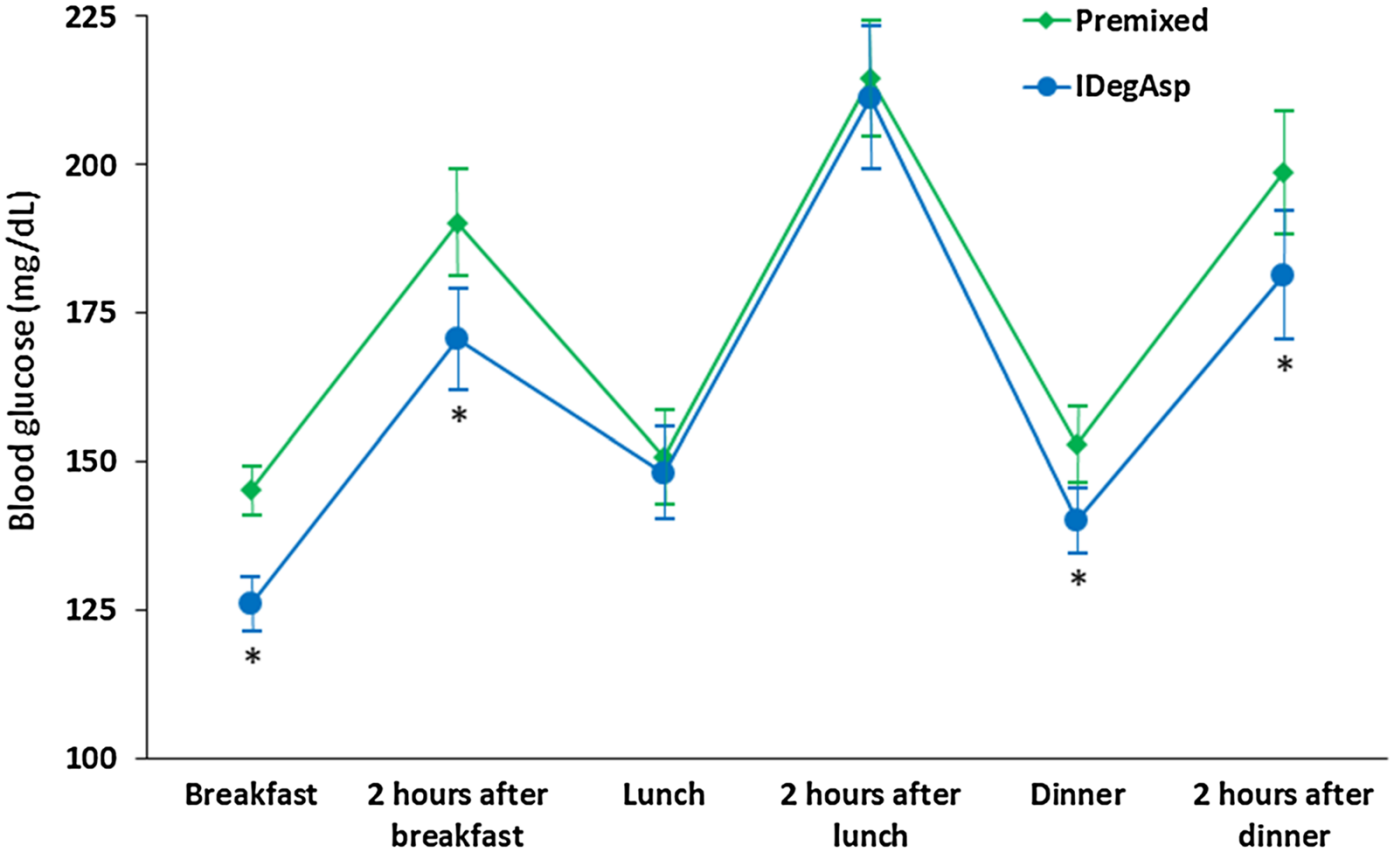
Mean before- and after-meal blood glucose levels following premixed insulin and IDegAsp use during the evaluation periods. Data are presented as means ± standard errors. Asterisks denote significant differences between treatments. *IDegAsp* insulin degludec/insulin aspart

Figure 2 illustrates all mean before- and after-meal blood glucose levels. Compared to the premixed insulin phase, the before-breakfast (145.3 ± 4.1 mg/dL vs. 126.0 ± 4.4 mg/dL; *P *< 0.001), after-breakfast (190.3 ± 9.0 mg/dL vs. 170.7 ± 8.5 mg/dL; *P *= 0.002), before-dinner (153.0 ± 6.5 mg/dL vs. 140.1 ± 5.4 mg/dL; *P *= 0.015), and after-dinner glucose levels (198.7 ± 10.3 mg/dL vs. 181.4 ± 10.7 mg/dL; *P *= 0.036) were lower in the IDegAsp phase. However, the before-lunch (150.8 ± 8.0 mg/dL vs. 148.2 ± 7.7 mg/dL; *P *= 0.657) and after-lunch glucose levels (214.7 ± 9.7 mg/dL vs. 211.4 ± 11.9 mg/dL; *P *= 0.616) did not significantly differ between phases.

A sub-group analysis of prior insulin type did not reveal significant differences in the SDs of the morning fasting blood glucose level (BHI30: 17.7 ± 2.1 mg/dL vs. 17.4 ± 1.7 mg/dL, *P *= 0.420 and BIAsp30: 22.6 ± 3.3 mg/dL vs. 21.6 ± 1.9 mg/dL, *P *= 0.741). Additionally, the M-value did not change significantly in either sub-group (BHI30: 42.0 [20.9, 67.9] vs. 25.9 [16.3, 49.5], *P *= 0.110 and BIAsp30: 62.1 [22.8, 108.0] vs. 30.1 [6.9, 89.0], *P *= 0.652). Finally, the mean blood glucose level was significantly lower in the IDegAsp phase than in the premixed insulin phase, irrespective of the insulin type (BHI30: 170.5 ± 7.0 mg/dL vs. 159.3 ± 6.0 mg/dL, *P *= 0.047 and BIAsp30: 181.4 ± 9.4 mg/dL vs. 167.4 ± 10.2, *P *= 0.027).

The QOL analysis suggested that switching the type of insulin therapy had a partial effect on the patients’ QOL (Table 3). Regarding the ITR-QOL questionnaire, the total and therapy-related feeling scores favored IDegAsp, whereas the social activity, physical activity, and daily activity scores did not significantly differ. Regarding the ITR-QOLN questionnaire, the score for glycemic control before breakfast improved with IDegAsp, whereas the total and other subscale scores (anxiety before sleep, disturbances during sleep, and well-being) did not significantly differ. Based on the correlation analysis, total ITR-QOLN score improvement was positively related to a reduction in SD of the morning fasting blood glucose level (r = 0.48, *P* = 0.004), whereas total ITR-QOL score showed no correlation (r = 0.50, *P* = 0.104). Changes in other endpoint variables such as the M-value and mean blood glucose level were not related to both ITR-QOL and ITR-QOLN score improvement (all *P* > 0.05).

**Table 3:** Changes in the scores of quality of life questionnaires related to insulin use

QOL score	Premixed insulin (n = 22)	IDegAsp (n = 22)	*P*-value
ITR-QOL score	107.5 [102.3, 112.8]	112.5 [107.3, 114.8]	0.002
Social activity	24.5 [23.0, 25.0]	25.0 [24.3, 25.0]	0.177
Physical activity	19.0 [16.3, 20.0]	20.0 [19.0, 20.0]	0.201
Daily activity	14.0 [13.0, 15.0]	15.0 [14.0, 15.0]	0.157
Therapy-related feeling	51.0 [50.0, 53.8]	54.0 [52.0, 55.0]	< 0.001
ITR-QOLN score	116.0 [111.0, 123.5]	121.0 [117.0, 122.7]	0.188
Disturbance during sleep	42.0 [41.7, 42.0]	42.0 [41.7, 42.0]	1.000
Anxiety before sleep	40.0 [37.0, 42.0]	38.5 [37.0, 42.0]	0.827
Well-being	22.5 [20.2, 23.7]	24.0 [22.5, 24.0]	0.241
Glycemic control before breakfast	14.5 [13.2, 17.7]	18.0 [15.0, 18.0]	0.011

Data are presented as median and interquartile ranges

*QOL* quality of life, *IDegAsp* insulin degludec/insulin aspart, *ITR-QOL* Insulin Therapy Related Quality of Life, *ITR-QOLN* Insulin Therapy Related Quality of Life at Night

According to a sub-group analysis stratified by renal function, the subjects with estimated glomerular filtration rate (eGFR) ≥ 60 mL/min/1.73 m^2^ (n = 13) had better M-value with IDegAsp than premixed insulin (60.2 [31.7, 76.9] vs. 28.9 [17.8, 49.5], *P *= 0.009), whereas those with eGFR < 60 mL/min/1.73 m^2^ (n = 9) had identical M-value in both therapies (36.3 [9.2, 110.4] vs. 23.6 [7.9, 101.8], *P *= 0.820). In contrast, the SDs of the morning fasting blood glucose level, ITR-QOL score, and ITR-QOLN score did not change significantly in either renal function group (all *P* > 0.05).

## Discussion

This is the first study to investigate changes in day-to-day glucose variability and QOL upon switching from premixed insulin to IDegAsp in a real-world setting. Twice-daily IDegAsp effectively improved both the morning and evening glucose levels and slightly improved the within-day glucose variability and daily QOL of patients previously treated with premixed insulin. However, no significant differences in day-to-day glucose variability during morning fasting were observed between phases.

The improved daily glucose variability observed with IDegAsp might be attributable to the stable glucose-lowering effect of the insulin degludec component [11, 12], which generally leads to strict titration with less fear of nocturnal hypoglycemia. This improvement emerged especially in patients with good renal function in this study, indicating that decreased renal function would lead to glucose variability. As day-to-day glycemic variability had also been associated with increased mortality [13], we measured our subjects’ fasting glucose levels every morning throughout the study periods to determine day-to-day variability. Although the results of the low day-to-day glucose variability of insulin degludec when compared with other forms of insulin [14] predicted the same results of the low day-to-day glucose variability with IDegAsp, there was no effect on lowering the day-to-day glucose variability in IDegAsp in the present study. Furthermore, contrary to our expectations, the “high” degree of resuspension had a better influence on low day-to-day glucose variability with IDegAsp (the various degrees of insulin resuspension in our study were similar to those of a previous report) [4]. These results might be attributable to the small study sample size and different glycemic targets used in our study. However, we observed a lower fasting blood glucose level with IDegAsp, compared with BIAsp30 or BHI30, without an increase in the incidence of hypoglycemia.

In our real-world medical setting, we often observe insulin intensification rather than insulin initiation. A patient’s psychological barriers to insulin intensification include perceived limitations of daily activities and an increased burden of injections [15]. Insulin therapy may have both positive and negative impacts on the QOL. In contrast to previous studies, which did not find that changes in the type of insulin therapy significantly impacted patients’ QOL [16], the present study found that the QOL related to insulin therapy improved significantly by changing insulin therapies. The flexibility of injection timing and lack of resuspension required during IDegAsp therapy might explain the improvements in the total and therapy-related feeling subscores in the ITR-QOL questionnaire. Another possible reason for QOL improvement included that improved glycemic control could obviously result in good QOL or the “new” insulin could potentially yield a good effect on QOL. Data from the ITR-QOLN questionnaire further suggested that uncontrollable hypoglycemia, especially nocturnal hypoglycemia, was a major concern among insulin users and that IDegAsp slightly reduced this concern. IDegAsp might potentially increase confidence with the expected low incidence of midnight hypoglycemia and low glycemic variability due to the long duration of the insulin degludec component with the lack of a “shoulder effect.” The positive correlation between total ITR-QOLN score improvement and day-to-day glycemic variably reduction in this study could support this increased confidence in IDegAsp among patients. However, the total ITR-QOLN score did not change, suggesting that a switch to IDegAsp might have a limited ability to improve nighttime confidence. The lack of significant changes in some of the QOL questionnaire subscores might also be explained by the nearly full marks assigned by users and an identical frequency of hypoglycemia with both therapies in our study, which differed from a previous analysis reporting hypoglycemia frequency [17].

Previous studies reported lower insulin doses with IDegAsp compared to BIAsp30 [6, 7]. In our study, we found that only the evening insulin dose decreased slightly with IDegAsp. This discrepancy between studies may be attributable to the relatively small insulin dose and different titration protocol used in our study. The decreased evening insulin dose did not affect HDL cholesterol level significantly, though insulin therapy was reported to have favorable effect on HDL cholesterol metabolism [18]. Regarding insulin characteristics, the insulin dose titrations with IDegAsp and premixed insulin should not be identical. According to the IDegAsp titration protocols used in previous studies [6, 7], the evening dose was adjusted based on the morning glucose levels, similar to the general titration of premixed insulin. However, this titration may have led to a continuous increase in the insulin dose with consequent hypoglycemia because the fasting glucose levels were mostly regulated by the total morning and evening insulin degludec component of IDegAsp and were therefore inadequate. In our study, the patients’ self-adjusted insulin doses adhered to the same fixed ratio for the morning and evening doses. Titration of the insulin dose with twice-daily IDegAsp should be conducted with careful attention to an adequate balance and a full understanding of the effects.

This study was subject to some limitations. First, our subjects were prior users of BHI30 or BIAsp30. However, this heterogeneity did not influence the primary endpoint results because a sub-group analysis of the prior type of insulin therapy did not reveal significant differences in the SD of the fasting blood glucose levels in the morning. Second, we cannot exclude the possibility of unrecorded hypoglycemia because the frequency of this event was calculated based on self-measured blood glucose levels or the patients’ symptoms. Future studies should aim to address this limitation, as continuous glucose monitoring (CGM) can help to detect potential hypoglycemic events. Third, day-to-day glucose variability was only evaluated using the before-breakfast glucose levels. However, the timing of the before-breakfast measurement must be optimal when evaluating differences between IDegAsp and premixed insulin therapies in terms of these characteristics. Furthermore, we used only the M-value to evaluate daily glucose variability because of the study feasibility; thus, studies involving CGM are needed. Finally, because this study lacked a cross-over design and had a small sample size and short study period, the switch in insulin regimen could not explain all the changes in endpoints; therefore, other factors such as lifestyle changes and physicians’ motivations might have contributed to these results. Longer-term prospective cross-over studies with a large number of subjects are needed to confirm the true differences between the insulin regimens, especially of hypoglycemia frequency.

## Conclusions

While there are possibilities of not reducing day-to-day glucose variability and afternoon glucose levels, IDegAsp can control morning and nocturnal glucose levels with improved daily glucose variability. Our findings suggest that IDegAsp can be useful for insulin intensification with effective glycemic control, an improved QOL, mealtime flexibility, and simplicity of use in patients treated with premixed insulin.

## Abbreviations

BHI30: human insulin 30%/neutral protamine Hagedorn insulin 70%
BIAsp30: insulin aspart 30%/protaminated insulin aspart 70%
IDegAsp: insulin degludec/insulin aspart
QOL: quality of life
SMBG: self-measured blood glucose
ITR-QOL: Insulin Therapy Related Quality of Life
ITR-QOLN: Insulin Therapy-Related Quality of Life at Night
eGFR: estimated glomerular filtration rate
CGM: continuous glucose monitoring

## References

[1] Inzucchi SE, Bergenstal RM, Buse JB, Diamant M, Ferrannini E, Nauck M (2015). Management of hyperglycemia in type 2 diabetes, 2015: a patient-centered approach: update to a position statement of the American Diabetes Association and the European Association for the Study of Diabetes. Diabetes Care.

[2] Freemantle N, Balkau B, Danchin N, Wang E, Marre M, Vespasiani G (2012). Factors influencing initial choice of insulin therapy in a large international non-interventional study of people with type 2 diabetes. Diabetes Obes Metab.

[3] Heise T, Eckers U, Kanc K, Nielsen JN, Nosek L (2008). The pharmacokinetic and pharmacodynamic properties of different formulations of biphasic insulin aspart: a randomized, glucose clamp, crossover study. Diabetes Technol Ther..

[4] Jehle PM, Micheler C, Jehle DR, Breitig D, Boehm BO (1999). Inadequate suspension of neutral protamine Hagendorn (NPH) insulin in pens. Lancet.

[5] Heise T, Nosek L, Klein O, Coester H, Svendsen AL, Haahr H (2015). Insulin degludec/insulin aspart produces a dose-proportional glucose-lowering effect in subjects with type 1 diabetes mellitus. Diabetes Obes Metab.

[6] Fulcher GR, Christiansen JS, Bantwal G, Polaszewska-Muszynska M, Mersebach H, Andersen TH (2014). Comparison of insulin degludec/insulin aspart and biphasic insulin aspart 30 in uncontrolled, insulin-treated type 2 diabetes: a phase 3a, randomized, treat-to-target trial. Diabetes Care.

[7] Kaneko S, Chow F, Choi DS, Taneda S, Hirao K, Park Y (2015). Insulin degludec/insulin aspart versus biphasic insulin aspart 30 in Asian patients with type 2 diabetes inadequately controlled on basal or pre-/self-mixed insulin: a 26-week, randomised, treat-to-target trial. Diabetes Res Clin Pract.

[8] Schlichtkrull J, Munck O, Jersild M (1965). The M-valve, an index of blood-sugar control in diabetics. Acta Med Scand.

[9] Ishii H, Yamamoto T, Ohashi Y (2001). Development of insulin therapy related quality of life measure (ITR-QOL). J Jpn Diabetes Soc..

[10] Ishii H, Furuya M, Iburi T (2008). Insulin therapy related QOL at night (ITR-QOLN). J Jpn Diabetes Soc..

[11] Heise T, Hermanski L, Nosek L, Feldman A, Rasmussen S, Haahr H (2012). Insulin degludec: four times lower pharmacodynamic variability than insulin glargine under steady-state conditions in type 1 diabetes. Diabetes Obes Metab.

[12] Heise T, Nørskov M, Nosek L, Kaplan K, Famulla S, Haahr HL (2017). Insulin degludec: lower day-to-day and within-day variability in pharmacodynamic response compared with insulin glargine 300 U/mL in type 1 diabetes. Diabetes Obes Metab.

[13] Muggeo M, Zoppini G, Bonora E, Brun E, Bonadonna RC, Moghetti P (2000). Fasting plasma glucose variability predicts 10-year survival of type 2 diabetic patients: the Verona Diabetes Study. Diabetes Care.

[14] Aso Y, Suzuki K, Chiba Y, Sato M, Fujita N, Takada Y (2017). Effect of insulin degludec versus insulin glargine on glycemic control and daily fasting blood glucose variability in insulin-naïve Japanese patients with type 2 diabetes: I’D GOT trial. Diabetes Res Clin Pract.

[15] Kunt T, Snoek FJ (2009). Barriers to insulin initiation and intensification and how to overcome them. Int J Clin Pract Suppl..

[16] Ishii H, Terauchi Y, Jinnouchi H, Taketsuna M, Takeuchi M, Imaoka T (2013). Effects of insulin changes on quality of life and glycemic control in Japanese patients with type 2 diabetes mellitus: the insulin-changing study intending to gain patients’ insights into insulin treatment with patient-reported health outcomes in actual clinical treatments (INSIGHTs) study. J Diabetes Investig..

[17] Christiansen JS, Niskanen L, Rasmussen S, Johansen T, Fulcher G (2016). Lower rates of hypoglycemia during maintenance treatment with insulin degludec/insulin aspart versus biphasic insulin aspart 30: a combined analysis of two Phase 3a studies in type 2 diabetes. J Diabetes..

[18] Aslan I, Kucuksayan E, Aslan M (2013). Effect of insulin analog initiation therapy on LDL/HDL subfraction profile and HDL associated enzymes in type 2 diabetic patients. Lipids Health Dis..

